# Biochemical characterization and anti-inflammatory properties of an isothiocyanate-enriched moringa (*Moringa oleifera*) seed extract

**DOI:** 10.1371/journal.pone.0182658

**Published:** 2017-08-08

**Authors:** Asha Jaja-Chimedza, Brittany L. Graf, Charlotte Simmler, Youjin Kim, Peter Kuhn, Guido F. Pauli, Ilya Raskin

**Affiliations:** 1 Department of Plant Biology, Rutgers University, New Brunswick, New Jersey, United States of America; 2 Center for Natural Product Technologies, Department of Medicinal Chemistry and Pharmacognosy, University of Illinois at Chicago, Chicago, Illinois, United States of America; 3 Nutrasorb, LLC, Freehold, New Jersey, United States of America; Institute of Biochemistry and Biotechnology, TAIWAN

## Abstract

*Moringa oleifera* Lam. is a tropical plant, used for centuries as food and traditional medicine. The aim of this study was to develop, validate and biochemically characterize an isothiocyanate-enriched moringa seed extract (MSE), and to compare the anti-inflammatory effects of MSE-containing moringa isothiocyanate-1 (MIC-1) with a curcuminoid-enriched turmeric extract (CTE), and a material further enriched in its primary phytochemical, curcumin (curcumin-enriched material; CEM). MSE was prepared by incubating ground moringa seeds with water to allow myrosinase-catalyzed enzymatic formation of bioactive MIC-1, the predominant isothiocyanate in moringa seeds. Optimization of the extraction process yielded an extract of 38.9% MIC-1. Phytochemical analysis of MSE revealed the presence of acetylated isothiocyanates, phenolic glycosides unique to moringa, flavonoids, fats and fatty acids, proteins and carbohydrates. MSE showed a reduction in the carrageenan-induced rat paw edema (33% at 500 mg/kg MIC-1) comparable to aspirin (27% at 300 mg/kg), whereas CTE did not have any significant effect. *In vitro*, MIC-1 at 1 μM significantly reduced the production of nitric oxide (NO) and at 5 μM, the gene expression of LPS-inducible nitric oxide synthase (iNOS) and interleukins 1β and 6 (IL-1β and IL-6), whereas CEM did not show any significant activity at all concentrations tested. MIC-1 (10μM) was also more effective at upregulating the nuclear factor (erythroid-derived 2)-like 2 (Nrf2) target genes NAD(P)H:quinone oxidoreductase 1 (NQO1), glutathione *S*-transferase pi 1 (GSTP1), and heme oxygenase 1 (HO1) than the CEM. Thus, in contrast to CTE and CEM, MSE and its major isothiocyanate MIC-1 displayed strong anti-inflammatory and antioxidant properties *in vivo* and *in vitro*, making them promising botanical leads for the mitigation of inflammatory-mediated chronic disorders.

## Introduction

Inflammation is a host defense mechanism to protect against pathogens, stresses and tissue damage, and is a major factor in the progression of many chronic diseases including ulcerative colitis, diabetes, atherosclerosis and arthritis [1]. The inflammatory response involves a combination of different signaling elements such as cytokines, nitric oxide (NO) and two key transcription factors, nuclear factor-kappa B (NF-κB) and nuclear factor (erythroid-derived 2)-like 2 (Nrf2) [2,3]. NO is an important inflammatory mediator produced by the nitric oxide synthases (NOSs), including the cytokine-inducible isoform (iNOS), and is implicated in the pathogenesis of chronic inflammatory diseases [4]. iNOS can be regulated by NF-κB, a major mediator of the cytokine-inducible inflammatory response, which also regulates the pro-inflammatory cytokines interleukins-1β and -6 (IL-1β, IL-6) and tumor necrosis factor α (TNF-α) [5]. Nrf2 activates multiple antioxidant and chemoprotective genes while inhibiting inflammatory signaling [6,7]. Exposure to chemical or environmental stresses causes Nrf2 to accumulate and translocate to the nucleus where it binds to the antioxidant response element (ARE) inducing the transcription of multiple target genes, including phase II detoxification enzymes such as NAD(P)H:quinone oxidoreductase 1 (NQO1), heme oxygenase 1 (HO1) and glutathione *S*-transferase (GST) [8]. Increased production of inflammatory mediators and reactive oxygen species (ROS) are implicated in many diseases and are, therefore, important targets for the treatment of inflammatory and oxidative stress mediated conditions [9,10]. Conventional medical treatment of many inflammatory diseases typically involves the use of nonsteroidal anti-inflammatory drugs (NSAIDs), however these medications can pose serious health risks including cardiovascular, renal and gastrointestinal complications [11].

**Fig 1:**
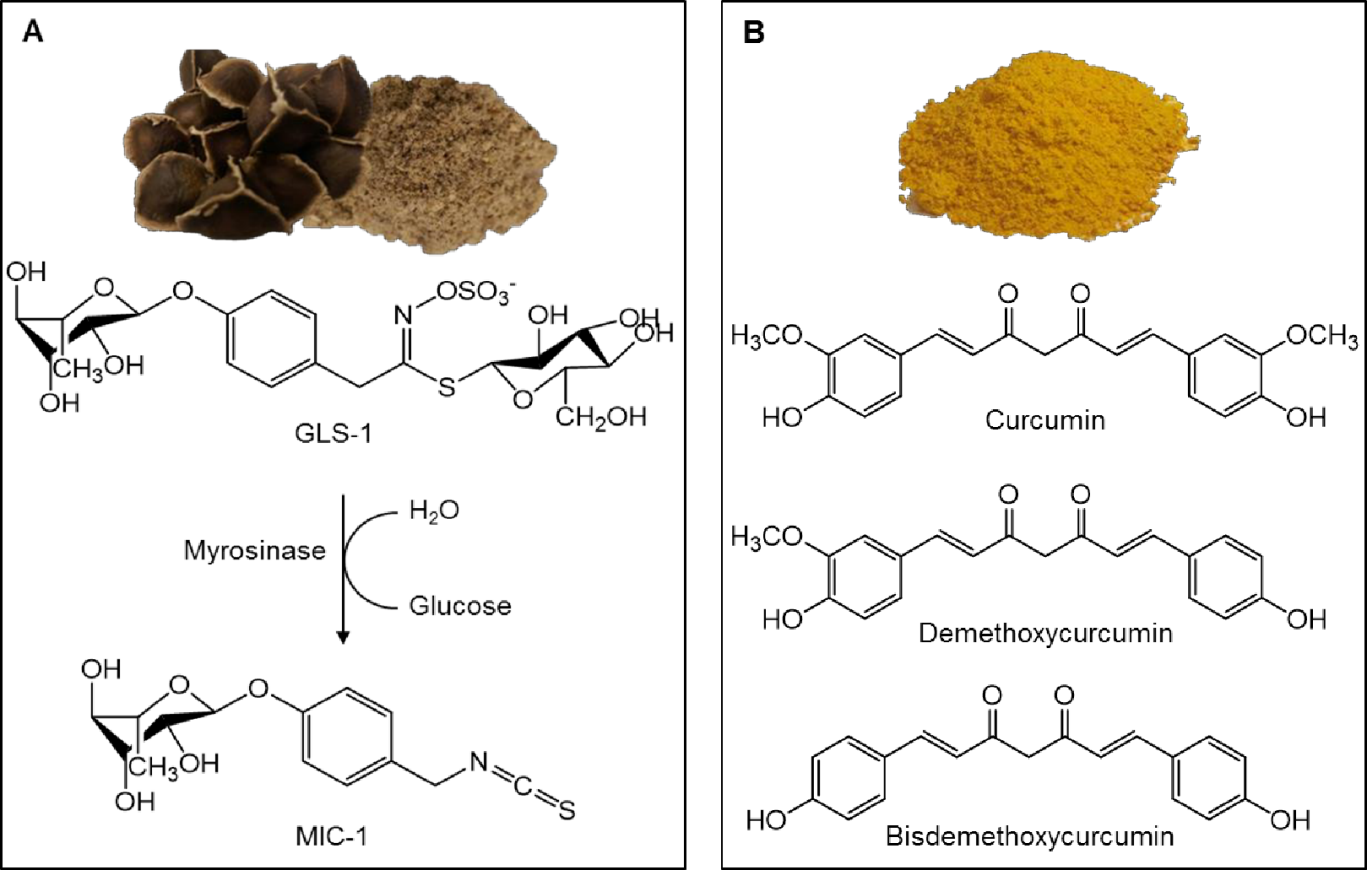
Chemical structures of the major phytoconstituents from *Moringa oleifera* (moringa) and *Curcuma longa* (turmeric). A) Moringa seeds and MSE prepared from them and the *in situ* bioconversion of GLS-1 to the designated bioactive, MIC-1. B) *Curcuma longa* extract and chemical structures of its three major curcuminoid constituents, curcumin (diferuloylmethane), demethoxycurcumin and bisdemethoxycurcumin.

Moringa (*Moringa oleifera* Lam.), a tropical tree native to Asia, and cultivated in Africa and both Central and South America, is one of about thirteen species belonging to the Moringaceae family (Order: Brassicales). Historically, all parts of the plant have been consumed as food and/or used in traditional medicine for the mitigation of inflammatory-mediated ailments, including cardiovascular and gastrointestinal diseases [12] and an increasing number of scientific studies support these traditional uses [13]. Though all parts of the moringa plant have been used traditionally, moringa seeds have specifically been reported to possess anti-inflammatory, antioxidant, hypotensive, antibacterial and chemopreventive properties [12,14,15]. Moringa seed-derived phytochemicals associated with these bioactivities include the unique glycosidic glucosinolates (GLSs), isothiocyanates (ITCs), nitriles, carbamates and thiocarbamates [14–16]. GLSs, stored in the seeds and other parts of the plant, undergo enzymatic conversion by the enzyme myrosinase (a *β*-thioglucosidase), forming an unstable intermediate, which, depending on the conditions, forms ITCs or nitriles that can be further converted to carbamates and thiocarbamates [17,18]. Four types of GLSs are found in the moringa plant, 4-[(α-L-rhamnosyloxy)-benzyl] glucosinolate (GLS-1, also referred to as glucomoringin) and three acetylated isomers (GLS 2–4), though only GLS-1 is reported in the seeds [18,19]. Upon wounding of the plant tissue, GLS-1 is converted to 4-[(α-L-rhamnosyloxy)-benzyl] isothiocyanate (MIC-1; Fig 1A), the proposed bioactive in the moringa seed extract (MSE) used in this study.

Moringa ITCs are structurally similar to ITCs derived from cruciferous vegetables of the Brassicaceae family (Order: Brassicales), such as sulforaphane from broccoli and phenethyl isothiocyanate (PEITC) from watercress. However, moringa ITCs are more stable than cruciferous ITCs due to the presence of the sugar moiety (Fig 1) [20]. Plant-derived ITCs are known for their antioxidant and anti-inflammatory effects [21], which are likely mediated through the activation of Nrf2 and inhibition of NF-κB. Nrf2 plays a central role in activating multiple antioxidant and chemoprotective genes while inhibiting inflammatory signaling [6,7]. Previous studies have shown that moringa ITCs, derived from the leaves and fruits, attenuate cellular NO production and inhibit gene expression of iNOS, TNF-α, IL-1β and IL-6 in RAW 264.7 murine macrophages [22,23]. MIC-1 has also been reported to specifically inhibit NF-κB activity in nude mice more effectively than sulforaphane [20]. MIC-1 and one of its acetylated isomers were also shown to induce NQO1 enzyme activity as effectively as sulforaphane in Hepa1c1c7 cells, suggesting that MIC-1 acts as an Nrf2 activator [24].

The rhizome of the tropical plant turmeric (*Curcuma longa* L., Zingiberaceae, Order: Zingiberales) is traditionally used as a botanical remedy for inflammatory disorders and is rich in curcuminoids (Fig 1B). Curcumin may account for about 90% of the total curcuminoids, and it is widely reported to possess antioxidant and anti-inflammatory properties [25,26]. Marketed for antioxidant and immune health benefits, curcuminoid-enriched turmeric extracts (CTEs) are commercially available as dietary supplements. Reportedly, CTEs act through the arachidonic inflammatory system via mediation of different cytokines [26]. Curcumin, the major component of CTEs, has been reported to share inflammatory molecular targets with ITCs, including reduction of iNOS and inflammatory cytokine (i.e. IL-1β and IL-6) expression via NF-κB inhibition [27,28]. It has also been reported to activate Nrf2 and upregulate the gene expression and enzymatic activity of its downstream targets, including NQO1 and GST [29]. Though many *in vitro* and *in vivo* studies point to efficacy of *C*. *longa* derived preparations; clinical trials have been inconclusive with regard to clinical outcome and/or phytochemical definition. Curcumin has recently been recognized among the class of compounds, which tends to inflate the observed bioactivity over a range of assays [30,31]. Based on a comprehensive meta-analysis of the literature, curcumin has also been characterized as an improbable drug lead because of its chemical instability, poor pharmacokinetics, and highly variable quality of commercially available supply [31]. Despite these limitations, CTEs remain among the top-40 botanical dietary supplements in the United States market [32].

This work further investigates the anti-inflammatory effects of MSE and MICs [33] (S1 File) and compares them to those of both CTE and CEM, respectively. A one-step method was optimized to prepare MSE enriched in its primary ITC constituent, MIC-1, ranging from 35–50% w/w. We evaluated the anti-inflammatory efficacy of standardized MSE, and compared it to a characterized, commercially-available CTE *in vivo* using a carrageenan-induced rat paw edema model. Furthermore, a direct comparison of the anti-inflammatory and antioxidant effects of the primary bioactive component of each extract, MIC-1 and CEM, was performed in lipopolysaccharide (LPS)-stimulated RAW 264.7 murine macrophages by measuring NO production, inflammatory gene expression (iNOS, IL-1β, and IL-6) and antioxidant gene expression (NQO1, HO1, GSTP1, and NRF2).

## Materials and methods

### Optimization and preparation of MSE

Moringa seeds were obtained from the Jamaica Moringa Farmers’ Association (Kingston, Jamaica). A voucher specimen was prepared and submitted to the Rutgers University CHRB Chrysler Herbarium (Accession number: 146375). The preparation of MSE was optimized based on the degree of biotransformation of GLSs to ITCs *in situ*, in order to obtain the highest MIC-1 content. The optimization was performed prior to preparation of the extract for *in vivo* and *in vitro* studies. The development and optimization of the procedure for the preparation of MSE involved incubating ground seeds in water at a controlled temperature with constant agitation for a duration of time, after which ethanol was added at 4x the volume of water to arrest the myrosinase reaction (Table 1). Incubation temperatures were first evaluated at 25°C or 37°C using a solvent ratio of 1:4 (g seeds:mL H_2_O) and incubation time of 2 h. Then solvent ratio was evaluated at 1:2, 1:3, and 1:4 using an incubation temperature of 37°C and incubation time of 2 h. Incubation time was also evaluated at 0.5, 1 and 2 h using an incubation temperature of 37°C and a solvent ratio of 1:4. All experiments were performed in triplicates using 10 g of ground seeds. Seed/solvent slurries were then filtered through a Whatman filter paper, and filtrate was reduced in volume via rotary evaporation and dried via lyophilization. The freeze-dried extracts were weighed and analyzed for MIC-1 content by LC-MS (see below).

**Table 1:** Optimization of MSE for MIC-1 content.

Extraction Parameters	% Yield of MSE (w/w)	% MIC-1 in MSE(w/w)
*Incubation Temperature (°C)*^§^		
25	12.58 ± 2.16	29.87 ± 0.91
37	13.24 ± 0.30	39.54 ± 1.03***
*Solvent Ratio (g seeds*:*mL H*_*2*_*O)* ^Ŧ^		
1:2	11.27 ± 0.24^a^	39.34 ± 1.17^a/b^
1:3	12.73 ± 0.06^b^	40.09 ± 0.50^a^
1:4	13.23 ± 0.43^b^	38.37 ± 0.76^b^
*Incubation Time (h)* ^ϕ^		
0.5	12.08 ± 0.22^a^	25.66 ± 0.73^a^
1	12.43 ± 0.13^a^	28.84 ± 0.77^b^
2	13.17 ± 0.21^b^	36.67 ± 0.84^c^

^*§*^ Solvent ratio of 1:4 and incubation time of 2 h

^*Ŧ*^ Incubation temperature at 37°C and incubation time of 2 h

^*ϕ*^ Solvent ratio of 1:4 and incubation temperature of 37°C.

Statistical analysis was done within each optimization parameter and data are represented as mean ± SD (n = 3). For temperature, analysis was performed by the student’s *t* test

***p < 0.001. For solvent ratio and incubation time, analyses were performed by one-way ANOVA followed by Tukey’s *post hoc* test, with significance represented by different letters (*p* < 0.05).

A large batch of MSE, obtained using optimized extraction conditions (1 g seed powder:3 mL water at 37°C for 2 h with constant agitation, followed by addition of 4x volume of ethanol, filtered and dried), was produced and stored at -20°C and used for all subsequent studies. The extract was analyzed and MIC-1 quantified by LC-MS. Nutritional analysis and fatty acid composition of MSE was performed by NJ Feed Labs (Trenton, NJ).

### Purification of MIC-1

To purify MIC-1, freeze-dried MSE was resuspended in ethanol (200 mg/mL) and sonicated for 30 min. The resuspended extract was then filtered using a 0.2 μm filter prior to injection in the HPLC. MIC-1 was purified from the filtered extract using a semi-preparative reversed-phase high performance liquid chromatography system equipped with an ultraviolet detector (HPLC-UV; Waters) monitored at 222 nm. The solvent system used for elution was water (solvent A) and 0.1% acetic acid in acetonitrile (solvent B) on a Phenomenex Synergi Hydro-RP column (4 μm, 250 x 21.20 mm, 80 Å) at a flow rate of 10 mL/min. MIC-1 was eluted using a gradient with initial conditions of 70% solvent A and 30% solvent B for 5 min. Solvent B was increased to 100% over 25 min and maintained for 5 min, returning to initial conditions over 2 min with an 8 min equilibration between injections. MIC-1 (retention time, R_t_, of 13.7 min) was collected, dried by rotary evaporation and subsequent lyophilization. The freeze-dried material was stored at -20°C. Confirmation of MIC-1 identity was based on comparison of retention time, UV spectrum and MS data of previously prepared standard material [23]. The purity of MIC-1 was analyzed by HR-LCMS based on the peak areas from the UV and MS as well as by qHNMR (see below).

### LC-MS analysis of MSE and MIC-1

Liquid-chromatography mass spectrometry (LC-MS) analysis of MSE was performed to quantify MIC-1 in the extract and to identify other phytochemicals present in the extract using a Thermo Q Exactive Plus ESI-Orbitrap coupled with a Dionex Ultimate 3000 HPLC-UV system. MSE was resuspended in 80% ethanol, briefly sonicated, and filtered through 0.2μm filter before injection. Chromatographic separation was accomplished using a gradient of increasing acetonitrile in 0.1% acetic acid, starting from 5% acetonitrile for 2 min, increasing to 50% over 15 min followed by a ramp up to 95% over 5 min and holding for 3 min before returning to initial conditions over 2 min. A Phenomenex Kinetex C8 (2.6 μm, 100 x 2.10 mm, 100Å) column was used for the chromatographic separation. High-resolution mass spectrometric (HRMS) analysis was performed in negative mode using the following conditions: heated electrospray (HESI) probe at 400°C at 3.50 kV, capillary temperature of 275°C in data dependent mode over a scan range of 100–1000 m/z, with stepwise collision energy, NCE of 15, 30, and 45 V.

Purified MIC-1 (94.1% w/w purity with 5% w/w H_2_O) was used as the standard for the quantification of MIC-1 in MSE. UV peak areas at 222 nm from LC-MS injections were used to generate a five-point standard curve with the concentration ranging from 62.5–1000 μg/mL (y = 5808.5x - 59084, R^2^ = 1). MSE was resuspended in ethanol and filtered through a 0.2 μm syringe filter prior to injections (1 μL) on the LC-MS.

### ^1^H NMR fingerprinting of MSE, qHNMR and HiFSA of MIC-1

For determination of MIC-1 purity, quantitative ^1^H NMR (qHNMR) analyses were performed using 3,5-dinitrobenzoic acid, DNBA (Fluka, TraceCERT, purity P = 99.54% w/w lot # BCBH8381V) as internal calibrant (IC). The IC stock solution was prepared at 11.6 mM in DMSO-*d*_6._ A total of 3.36 mg of MIC-1 and 7.91 mg of MSE were diluted in 600 μL and 300 μL of the IC stock solution, respectively. From these preparations, 200 μL aliquots were transferred with calibrated glass pipets into 3 mm standard NMR tubes (Norell part no. S-3-HT-7, Norell Inc., Landisville, NJ). The 1D ^1^H NMR spectra were acquired at 298 K under quantitative conditions (qHNMR) using a 90° excitation pulse experiment (Bruker pulprog: zg), on a Bruker AVANCE 900 MHz equipped with a 5 mm CPTCI probe. The 90° pulse width for each sample was determined by prorating the measured 360° pulse width (p90 = 1/4 × p360). The probe was frequency tuned and impedance matched before each acquisition. For each sample, 32 scans (ns) and 4 dummy scans (ds) were recorded with the following parameters: pulse width (P1) of typically 10.65 μs (90°), spectral width of 25 ppm, relaxation delay (D1) of 60 s, receiver gain (RG) set to 32 for MIC-1 and 64 for MSE. The total duration of each ^1^H NMR acquisition was 32 min.

Off-line data processing was performed using the Mnova NMR software package (v.6.0.2, MestreLab Research S.L., A Coruña, Spain). ^1^H and ^13^C chemical shifts (δ) were expressed in ppm with reference to the residual solvent signal (DMSO-*d*_*5*_: ^1^H spectrum: 2.500 ppm). The following processing scheme was used: a mild Lorentzian-to-Gaussian window function (line broadening = −0.3 Hz, Gaussian factor = 0.01) was applied, followed by zero filling to 256k acquired data points before Fourier transformation. After manual phasing, a fifth order polynomial baseline correction was applied. The purity determination of MIC-1 was performed as described previously using both the 100% and the absolute (with IC = AIC) methods [34]. The freely available calculation spreadsheet were utilized for this purpose (see supporting information, and http://gfp.people.uic.edu/rt/qHNMRpurityassay.html#qHNMRcalculations). PERCH NMR software (v.2013.1, PERCH Solutions Ltd., Kuopio, Finland) was employed for the iterative ^1^H NMR full spin analysis (HiFSA) in order to accurately describe chemical shifts, but most importantly coupling constants, and individual line widths (*δ*, *J*, and *w*) for MIC-1 (spin parameter file “.pms” in supporting information). The HiFSA profiling of compounds has been described in previous articles [35,36]. The original 1D, ^1^H NMR data (FIDs) of MSE and MIC-1, results of the purity analysis, as well as the HiFSA profile of MIC-1 (as pms file), are made freely available at doi:10.7910/DVN/PRHUWB, (Harvard, Dataverse).

### Carrageenan-induced rat paw edema

Forty male Sprague-Dawley rats (126–150 g) were purchased from Charles River Laboratories (Malvern, PA) and acclimated for one week at 22 ± 2°C on a light/dark cycle of 12 h. Animals were housed two per cage and allowed access to food and water *ad libitum*. Experiments were carried out using the approved protocol by the Rutgers University Institutional Animal Care and Use Committee (Protocol # 05–037). All *in vivo* experiments using MSE were standardized to MIC-1 (based on 38.9% w/w MIC-1 in MSE). Animals were randomly assigned to one of 5 treatment groups: vehicle control (15% sodium carboxymethyl cellulose), aspirin at 300 mg/kg (purity < = 100%), MSE (at 250 or 500 mg/kg MIC-1), and CTE at 400 mg/kg (Nature’s Bounty, Inc., Bohemia, NY; analyzed to contain 68% curcuminoids as determined by qHNMR, S3 Fig). MSE dose was determined based on our pilot study which showed no toxicity up to a dose of 500 mg/kg. The dose used for CTE was based on previous studies showing efficacy up to 400 mg/kg in a similar model [37]. MSE and CTE were prepared in 15% carboxymethyl cellulose, which formed a suspension and was constantly stirred before and during dosing of the animals. Treatments were administered orally by gavage at 10 mL/kg body weight. All chemicals and reagents were obtained from Sigma-Aldrich (St. Louis, MO), unless noted otherwise.

Induction of inflammation was achieved based on the protocol by McCarson [38]. Briefly, the plantar region of the right hind paw of rats were injected with 100 μL of 1% w/v λ-carrageenan solution in saline 30 min after the animals were administered the different treatments. Paw volume was measured up to the natural hairline of the paw using a plethysmometer (IITC Life Science Inc., Woodland Hills, CA) before and after carrageenan injection hourly up to 4 h post injection. The extent of paw edema was determined using the following formula % paw edema = (V_t_−V_0_)/V_0_ x 100, where V_t_ is the paw volume at time points after carrageenan injection and V_0_ is the paw volume prior to carrageenan injection. The inhibition of paw edema was determined using the following formula: % inhibition = [(V_t_−V_0_) _control_—(V_t_−V_0_) _treatment_] / [(V_t_−V_0_) _control_] x 100.

### RAW 264.7 murine cell macrophage culture

For *in vitro* studies, MIC-1 was purified from MSE to evaluate its role as the primary phytochemical responsible for the anti-inflammatory effects and to compare its effects with curcumin (Sigma-Aldrich, St. Louis, MO; containing 73.0% w/w curcumin and 90.0% w/w curcuminoids as determined by qHNMR, S3 Fig). RAW 264.7 murine macrophages (ATCC, TIB-71) were used as described previously [23]. Cells were cultured in Dulbecco’s modified Eagles medium (DMEM) supplemented with 10% fetal bovine serum (FBS) and 1% penicillin/streptomycin, and incubated at 37°C in 5% CO_2_. For experiments, cells were subcultured by cell scraping and plated at a density of 4 x 10^5^ cells/mL in 24-well plates for 18 h until confluent. Prior to the treatment with test materials, cells were washed with PBS and replaced with fresh DMEM media, without FBS. Subsequently, cells were treated (in triplicates) as described below. All test material (MSE, MIC-1 and CEM) was dissolved in ethanol prior to treatment of the cells at stock concentrations of 200x the final concentration for each treatment. At least three independent experiments were performed for each study using different cell passages (p3–8).

### Cell viability (MTT assay)

Cell viability was determined using the MTT (3-(4,5-dimethylthiazol-2-yl)-2,5-diphenyltetrazolium bromide) assay. Cells were either left untreated (no-treatment control, NT), treated with 0.475% ethanol as a vehicle-treated control (Ctl), or treated with MIC-1 (1, 5 or 10 μM) or CEM (curcumin concentration of 0.7, 3.5 or 7 μM) for 8 h in DMEM without FBS as previously described [23]. MTT (100 μg/mL) was added to the cells 3 h prior to the end of incubation, after which the media was removed and DMSO was added to dissolve the formazan crystals. Absorbance was measured at 570 nm using a Synergy HT plate reader (Biotek, Winooski, VT). Data were normalized to Ctl, which was set as 100% viability.

### Nitric oxide (NO) assay

Cells were treated for 2 h with vehicle (0.475% ethanol), MIC-1 (pure) or MIC-1 (in MSE) at 0.05, 0.1, 0.5, 1, 5 or 10 μM), and MIC-1 (1, 5 or 10 μM) or CEM (curcumin concentration of 0.7, 3.5 or 7 μM). To stimulate the inflammatory response, LPS (1 μg/mL, Sigma, St. Louis, MO) was added, and the cells were incubated for an additional 6 or 22 h with the treatment compounds/extract. To determine the basal levels of NO production, a set of Ctl cells was not stimulated with LPS. At the end of the incubation period, NO in the cell media was quantified using the Promega Griess Reagent System (Promega Corporation, Madison, WI) according to the manufacturer’s instructions. Subsequently, cells were washed with PBS and frozen at -20°C for quantifying cellular total proteins. Total protein concentrations were measured using the Pierce BCA Protein Assay kit following the manufacturer’s instructions, and used for normalization of cellular NO production. Data were further normalized to LPS-stimulated (+LPS) control, which was set to 100% NO production.

### Gene expression

Cells were treated as described for the NO assay, after which cells were washed 2x in PBS, collected in TRIzol Reagent (Life Technologies, Carlsbad, CA, USA), and stored at -80°C for RNA extraction. Gene expression assays were performed as described previously [23]. Briefly, total RNA was extracted from macrophages in TRIzol Reagent and treated with Deoxyribonuclease I (Life Technologies) according to the manufacturer’s instructions. RNA quality was assessed on the NanoDrop 1000 (NanoDrop Technologies, Wilmington, DE, USA). cDNA synthesis was performed using the ABI High Capacity cDNA Reverse Transcription Kit (Applied Biosystems, Foster, City, CA) with RNAse I inhibitor, according to the manufacturer’s instructions, using 5 μg RNA as a template in a 25 μL reaction. For inflammatory markers analysis, cDNAs were diluted 25-fold for qRT-PCR analysis on the QuantStudio 3^®^ Real-Time PCR System (Applied Biosystems) with Power SYBR Green PCR master mix (Applied Biosystems) and primers were pre-validated as follows: β-ACTIN forward 5’-AAC CGT GAA AAG ATG ACC CAG AT-3’, reverse: 5’-CAC AGC CTG GAT GGC TAC GT-3’; iNOS forward 5’-CCC TCC TGA TCT TGT GTT GGA-3’, reverse 5’-TCA ACC CGA GCT CCT GGA A-3’; IL-1β forward 5’-CAA CCA ACA AGT GAT ATT CTC CAT- 3’, reverse 5’-GAT CCA CAC TCT CCA GCT GCA-3’; IL-6 forward 5’-TCG GAG GCT TAA TTA CAC ATG TTC-3’,reverse 5’ TGC CAT TGC ACA ACT CTT TTC T-3’. The thermal cycler profile was as follows: 2 min, 50°C; 10 min, 95°C; 15 s, 95°C; 1 min, 60°C for the dissociation stage; 15 s, 95°C; 1 min, 60°C; 15 s, 95°C for 40 cycles. For antioxidant marker analysis, cDNAs were diluted 3-fold with Taqman Fast Advanced Master Mix and inventoried Taqman primer sets (ThermoFisher Scientific) were used for analyses of GADPH (Mm99999915_g1), NQO1 (Mm01253561_m1), HO1 (Mm00516005_m1), GSTP1 (Mm04213618_gH), and NRF2 (Mm00477784_m1). Gene expression was quantified by the comparative ΔΔCt method and normalized to GADPH. Vehicle with LPS treatment served as the calibrator and was assigned a value of 1.0.

### Statistical analysis

For cell assay results, all data were analyzed by one-way analysis of variance (ANOVA) followed by Tukey’s or Dunnett’s *post hoc* test using GraphPad Prism 6.0 software (GraphPad, La Jolla, CA). Prior to ANOVA analysis of gene expression results, outliers were removed using the robust regression followed by outlier identification (ROUT) method, and normality of cleaned data was assessed using the Kolmogorov-Smirnov normality test in GraphPad. All other statistical analyses were performed using Minitab 17 software (Minitab, Inc., State College, PA). *P* < 0.05 was considered significant.

## Results

### Optimization and characterization of MSE

Preparation of isothiocyanate-enriched MSE was based on the *in situ* biotransformation of GLS-1 to MIC-1. The extraction process was optimized to generate a high-yield extract with high ITC content by manipulating the solvent ratio, incubation temperature, and incubation time (Table 1). An increased temperature from 25 to 37°C did not produce a significantly higher yield of MSE (12.6% to 13.2%), however the higher temperature significantly increased MIC-1 content in MSE from 29.9% to 39.5%. Increasing the volume of water added to ground seeds from 1:2 g:mL to 1:3 or 1:4 g:mL during the bioconversion process slightly improved MSE yield from 11.27% to 12.73% or 13.23%, and resulted in minor variations in MIC-1 content (38.37–40.09%). Increased incubation time from 0.5 h to 2 h significantly increased MSE yield from 12.08% to 13.17% and significantly increased MIC-1 content in MSE from 25.66% to 36.67% (a 43% improvement). MSE prepared using the optimized extraction procedure resulted in an extract containing 38.9% MIC-1 w/w. This extract was used in all subequent experiments.

**Fig 2:**
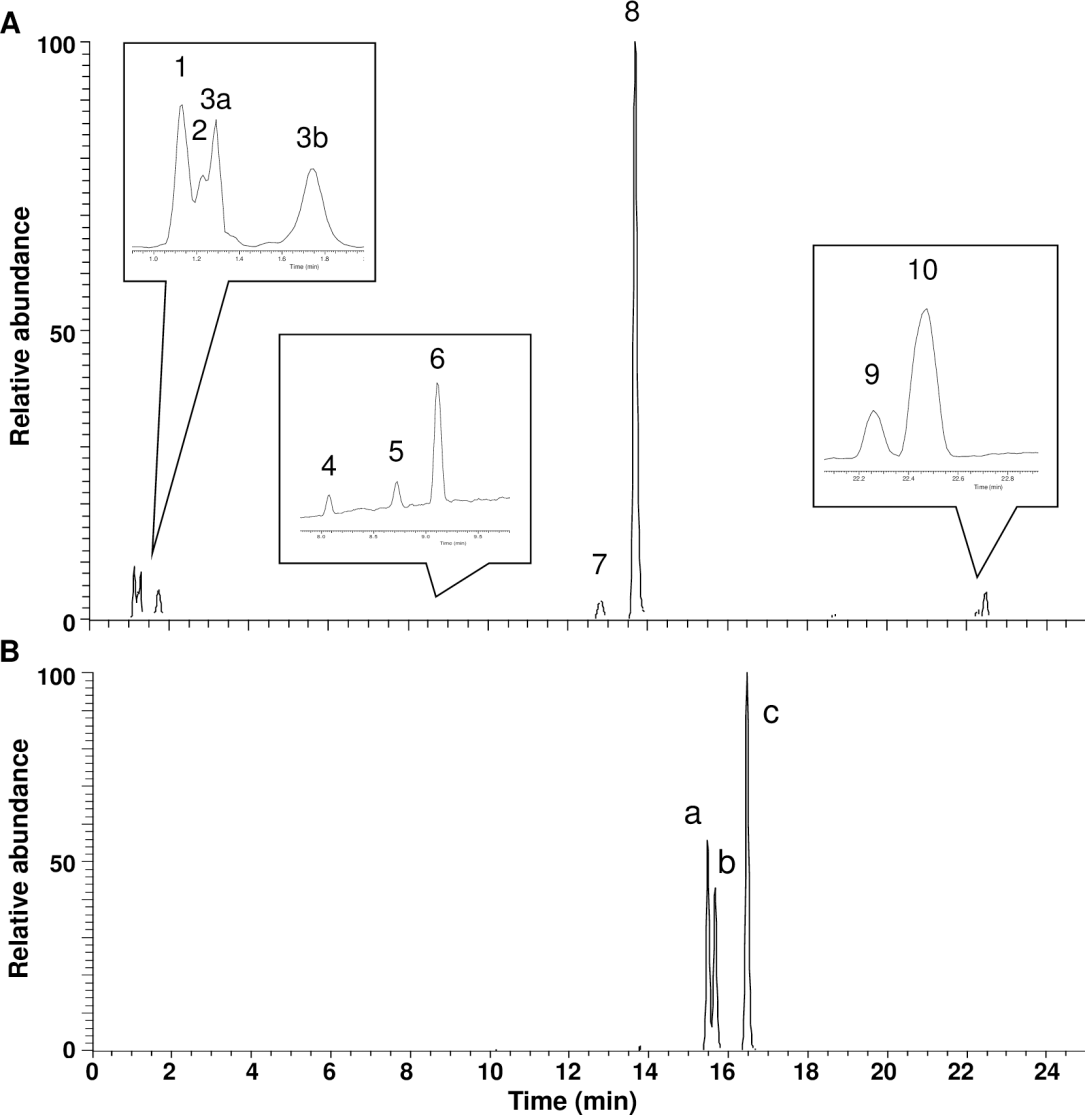
MS Chromatogram of MSE showing the phytochemical composition according to the detected molecular ions. A) Full spectrum of MSE. Identification of compounds was based on the molecular weight and MS/MS fragmentation pattern, and in some cases the UV profile, in conjunction with previous studies identifying different phytochemicals from moringa seeds. B) Extracted m/z 412 corresponding to the acetylated ITCs.

Proximate nutritional analysis of standardized and optimized MSE (Table 1 in S1 Table) resulted in a compositional profile of moisture (13.92%), protein (18.27%), fat (1.76%), fiber (0.60%), ash (4.01%), and carbohydrates (22.54%, of which 45.4% are sugars). The fatty acid profile (Table 2 in S1 Table) indicates that the most abundant fatty acids present were oleic acid, palmitic acid, behenic acid and stearic acid having relative abundances of 72.60%, 8.90%, 4.06% and 3.79% respectively. The phytochemical characterization of MSE was performed by LC-MS and analysis of MS/MS spectra. The chromatographic profile of MSE (Fig 2A) confirmed MIC-1 as the predominant component. Extraction of m/z 412.1081 [M+CH_3_COO^-^]^-^ resulted in three peaks (**a**, **b**, **c**, Fig 2B), corresponding to the acetylated ITCs previously identified in moringa leaves and fruits [22,23]. Peak assignments are listed in Table 2 and are based on the MS data and fragmentation pattern in agreement with previously identified phytochemicals found in moringa seeds. The following are tentative assignments of the major phytochemicals in MSE and their relative abundance obtained from the LC-MS chromatogram are indicated in brackets: **1**—sucrose (10), **2**—malic acid (5), **3a,b**—GLS-1 (5,5), **4a,b**—catechin/epicatechin and procyanidin dimer (overlapping peaks, <0.5), **5**—unknown (<0.5), **6**—niazirin (1), **7**—niazimicin (3), **8**—MIC-1 (100), **9**—palmitic acid and 2-hydroxy behenic acid (overlapping peaks, 2) and **10**—oleic acid (5).

**Table 2:** Identification of compounds in the extract based on the m/z.

Peak #	Retention time (mins)	*m/z* (Negative mode)	Observed fragment ions (Relative abundance)	Species	Peak assignment	Refs
1	1.13	341.1092 (683.2260 dimer)	179.0562, 119.0350, 113.0245, 101.0244, 89.0243(100), 71.0134, 59.0133	[M-H]^-^, [2M-H]^-^	Sucrose	
2	1.23	133.0144	115.0037(100), 71.0135	[M-H]^-^	Malic acid I	[39]
3a	1.29	570.0966	96.9601(100), 74.9906	[M-H]^-^	GLS-1	
3b	1.75	570.0966	96.9601(100), 74.9906	[M-H]^-^	GLS-1	
4a	8.06	289.0724	245.0819(100), 203.0714, 179.0350, 151.0401, 137.0244, 125.0244, 109.0295	[M-H]^-^	Catechin/epicatechin	
4b	8.06	577.1368	425.0875, 407.0781, 289.0717, 125.0245(100)	[M-H]^-^	Proanthocyanidin B-type dimer	
5	8.71	327.1093	121.0296(100), 59.0133	-	Unknown	
6	9.12	338.1253	132.0456, 59.0133(100)	[M-H]^-^	Niazirin	[40]
7	12.81	356.1179 (416.1389)	164.0177, 104.0176(100), 57.9751	[M-H]^-^, [M+CH_3_COO^-^]^-^	Niazimicin	[40]
8	13.69	370.0971	-	[M+CH_3_COO^-^]^-^	MIC-1	
9a	22.26	255.2334	-	[M-H]^-^	Palmitic acid	[41]
9b	22.26	355.3224	-	[M-H]^-^	2-Hydroxy behenic acid	
10	22.47	281.2490	-	[M-H]^-^	Oleic acid	[41]
a	15.48	412.1081	-	[M+CH_3_COO^-^]^-^	Ac-MIC*	
b	15.67	412.1081	-	[M+CH_3_COO^-^]^-^	Ac-MIC*	
c	16.46	412.1081	-	[M+CH_3_COO^-^]^-^	Ac-MIC*	

*Ac-MIC–Acetylated moringa ITCs [23]

**Fig 3:**
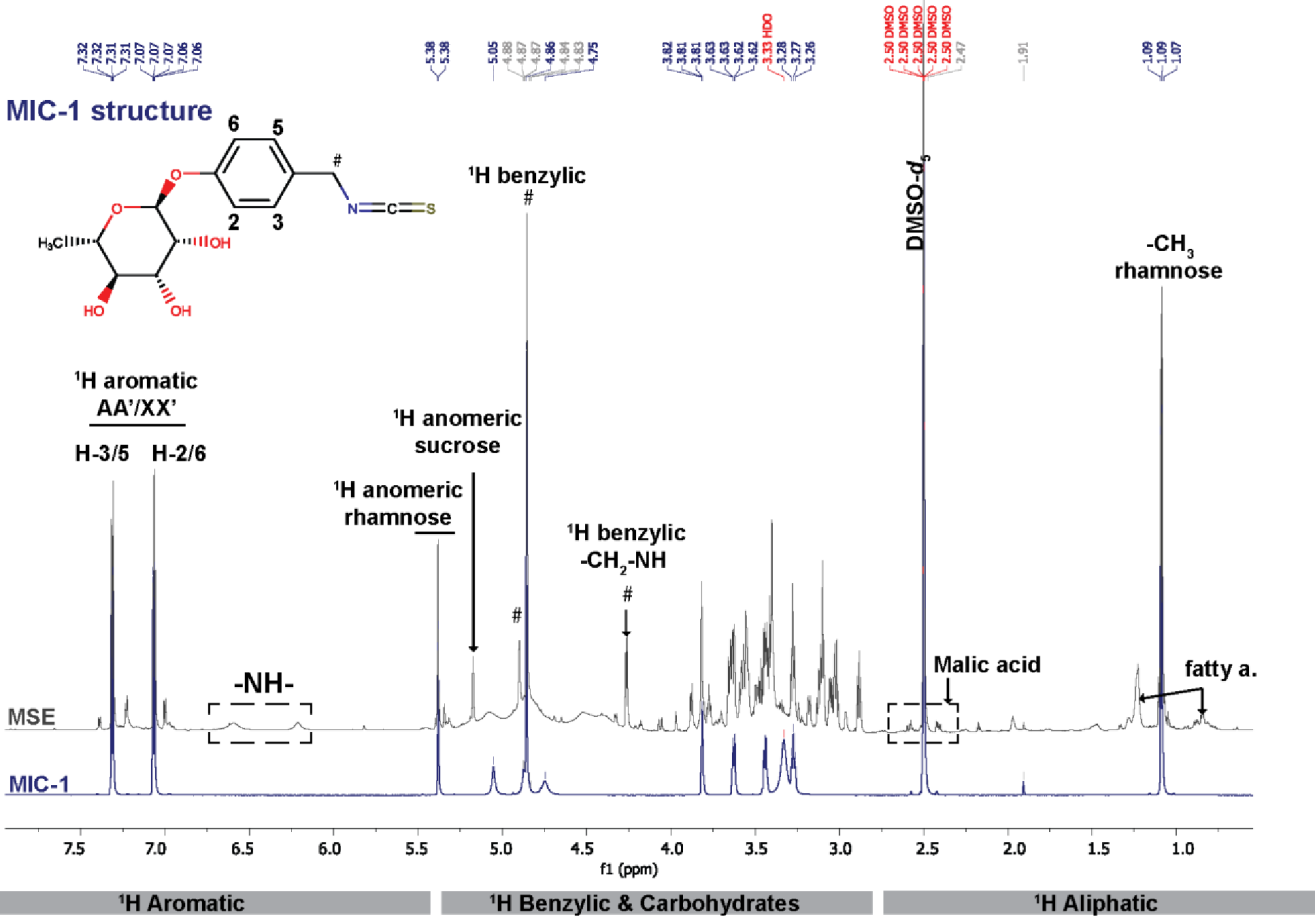
^1^H NMR fingerprinting of optimized MSE (900 MHz, DMSO-*d*_6_) compared with the purified MIC-1. Three major regions were identified in the ^1^H-NMR fingerprints of MSE, providing key structural information of compounds present in the crude extract. The results confirmed than MIC-1 was the most abundant compound, followed closely by congeneric MIC metabolites, sucrose, fatty acids, and malic acid, which were also detected by MS/MS.

Additionally, ^1^HNMR fingerprinting of MSE (Fig 3) was performed to complement the LC-MS profiling and to confirm the relative concentration of the most abundant compounds. The comparative ^1^H NMR spectra demonstrate that MIC-1 was the most abundant metabolite in the optimized MSE. The second most abundant metabolites were other closely related MIC-1 structures, as indicated by the presence of small characteristic aromatic protons (AA’XX’ system) on either side of the major ones (H-2/6 and H-3/5). Those signals could belong to either niazirin, niazimicin or a GLS-1 derivative (S2 Fig). Finally, the presence of sucrose (identified by its anomeric ^1^H), malic acid and a series of fatty acids was also confirmed by analysis of the ^1^H NMR spectrum, and comparison with reference spectra.

### *In vivo* anti-inflammatory activity

**Fig 4:**
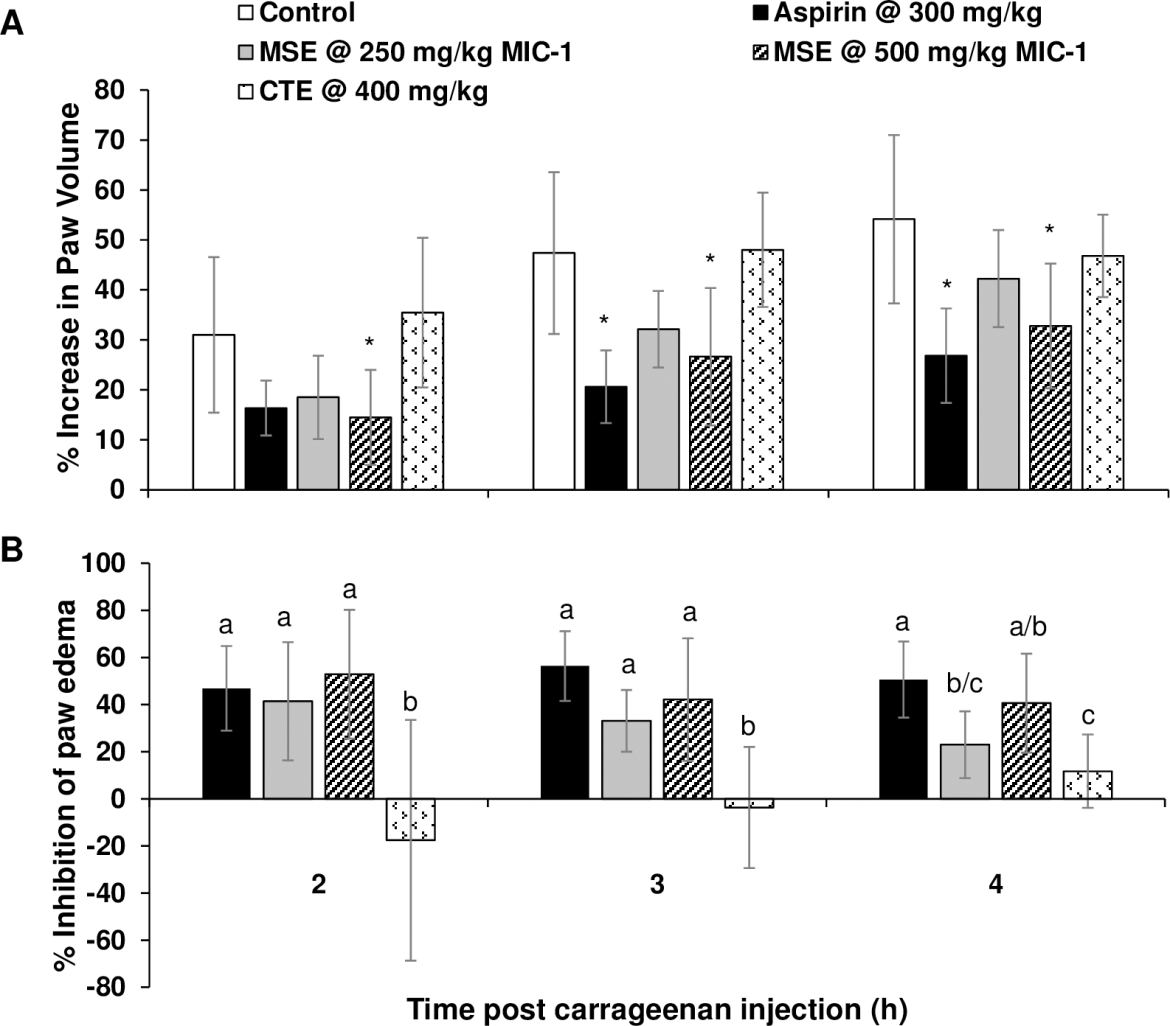
Inflammatory response in the hind-paw of rats treated with MSE and CTE prior to injection with carrageenan. A) Percent increase in paw volume. Statistical analysis was performed by one-way ANOVA followed by Dunnett’s *post-hoc* test. Bars showing an asterisk indicate significant difference from the vehicle control (*p* < 0.05). B) Percent inhibition of paw edema. Statistical analysis was performed by one-way ANOVA followed by Tukey’s *post-hoc* test. Significance is indicated by different letters (*p* < 0.05). Data are the mean ± SD (n = 7–8).

The carrageenan-induced rat paw edema model has been widely utilized for the identification of inflammatory effects of natural products and synthetic drugs. The model involves the development of acute inflammation in the right hind paw; maximum edema is observed within 2 to 4 h post carrageenan injection. Paw edema (paw volume) gradually increased in all five treatment groups for at least 4 h post carrageenan injection (Fig 4A). The negative control group (injected with carrageenan and gavaged with vehicle only) showed the largest increase in paw volume of 54% at 4 h compared to baseline. The aspirin-treated positive control group showed the lowest increase in paw volume of 27% at 4 h compared to baseline. In the animals treated with MSE, there was a dose dependent increase in the paw volume. MSE doses delivering 500 mg/kg MIC-1 and 250 mg/kg MIC-1 resulted in 33% and 42% increases in paw volume at 4 h following carrageenan injection, compared to the baseline. The CTE-treated group exhibited a 47% increase in the paw volume after 4 h. The aspirin group showed the greatest inhibition of paw edema of more than 40% between 2 h and 4 h (Fig 4B). The low dose MSE showed greater than 40% inhibition of paw edema at 2 h and a gradual reduction in inhibition to 23% at 4 h. The high dose group showed greater than 40% inhibition of paw edema at 2 h and 4 h, similar to the effect observed in the aspirin-treated group. The turmeric-treated group did not show inhibition of paw edema up to 3 h post carrageenan injection, however 11% inhibition was observed at 4 h, which was significantly lower than the level of inhibition observed in the aspirin and high MSE dose-treated groups.

### *In vitro* anti-inflammatory activity

#### NO production of MSE (containing MIC-1) and pure MIC-1

**Fig 5:**
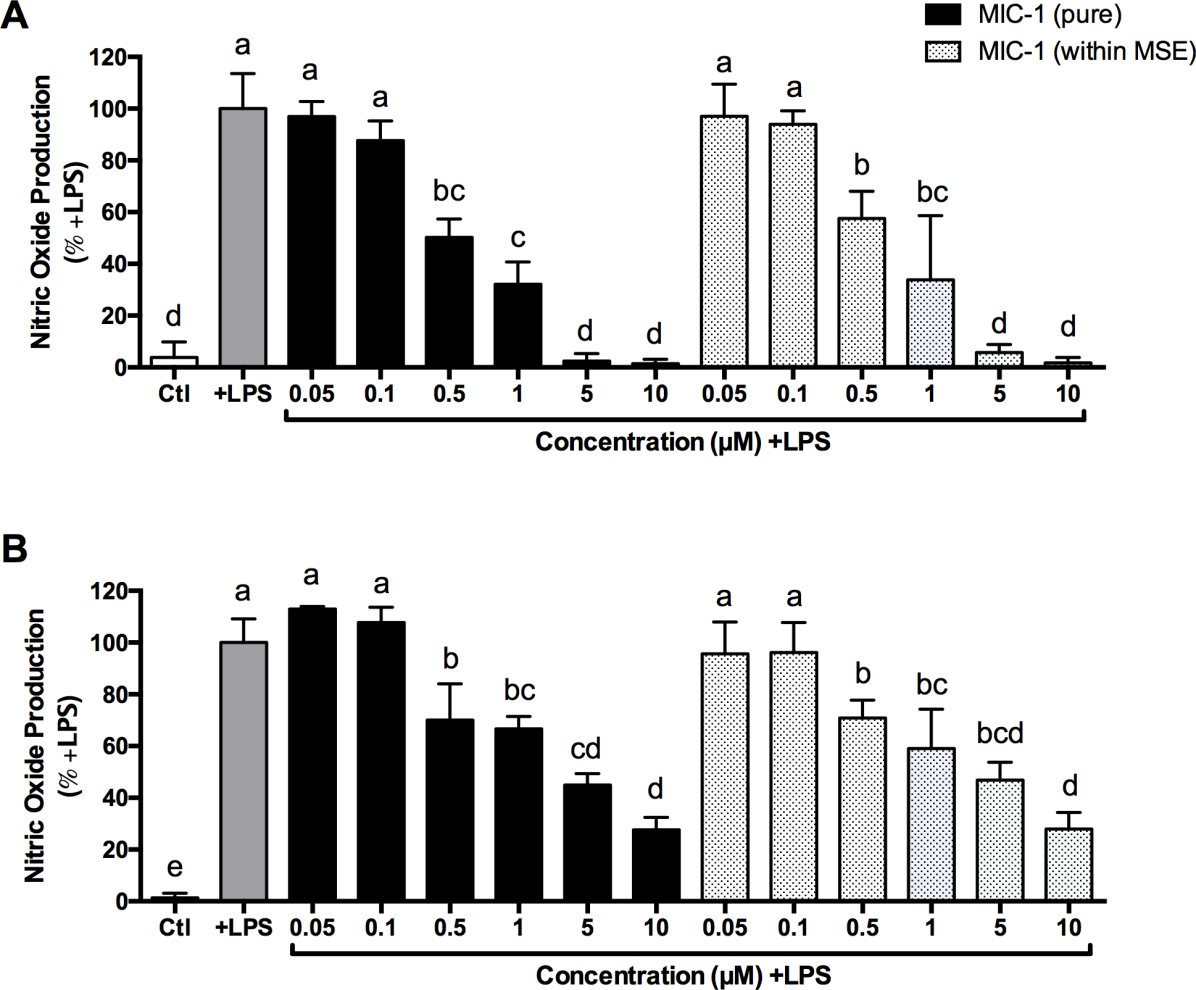
Effects of equivalent concentrations of MIC-1 (0.05, 0.5, 0.1, 1, 5, and 10 μM), delivered as a pure compound or as complex MSE, on NO production in LPS-stimulated RAW 264.7 murine macrophages. NO production in cells treated for A) 8 h and B) 24 h, compared to vehicle-treated control without LPS (Ctl) and LPS-stimulated control (+LPS). Data are the mean ± SD (n = 3–6). Different letters indicate significant differences, one-way ANOVA followed by Tukey’s *post hoc* test.

There was no significant difference observed in NO production in the LPS-stimulated murine macrophages treated with equivalent concentrations of MIC-1 delivered as a pure compound or as part of the complex MSE after 8 or 24 h. In the cells treated for 8 h, purified MIC-1 at concentrations of 0.05, 0.1, 0.5, 1, 5 and 10 μM reduced NO production by 3.3%, 12.4%, 49.8%, 67.9%, 97.5% and 98.6% respectively, compared to MSE at equivalent concentrations of MIC-1 with reductions of 3.0%, 6.1%, 42.4%, 66.1%, 94.3% and 98.2% respectively (Fig 5A). In the 24 h treatment, purified MIC-1 reduced NO production by -12.9%, -7.7%, 30.0%, 33.3%, 55.1% and 72.3%, whereas MSE (containing equivalent concentrations of MIC-1) reduced NO production by 4.3%, 3.9%, 29.1%, 40.9%, 53.1% and 72.1% (Fig 5B). Cells treated for 8 h showed the most significant reduction in NO production and this incubation time was used for all subsequent *in vitro* studies.

#### Cell viability and NO production

**Fig 6:**
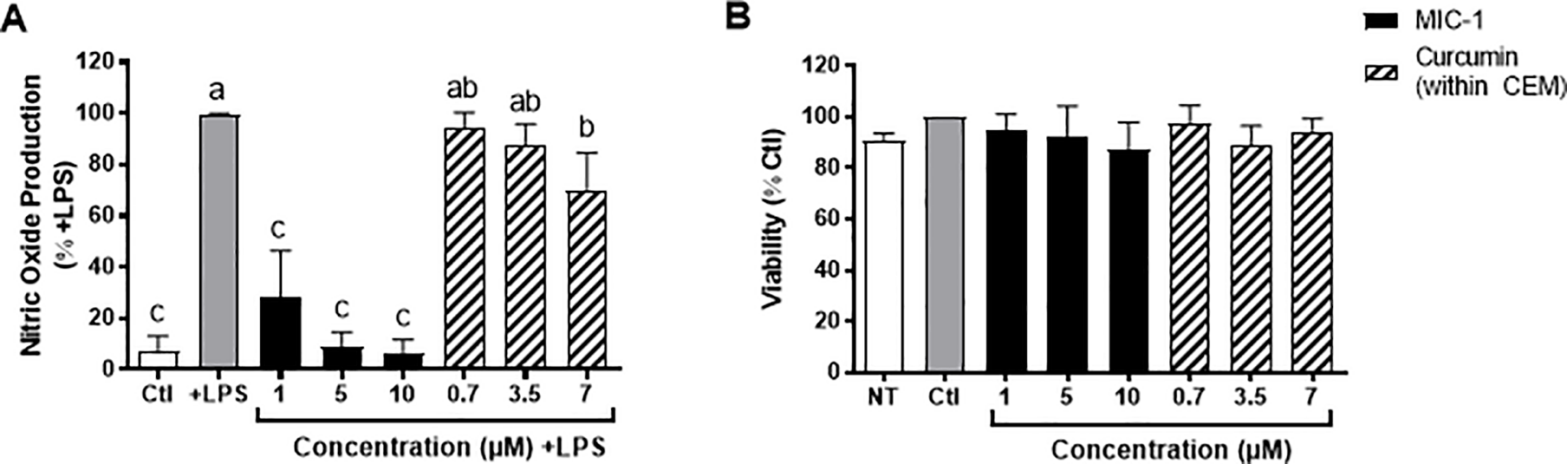
Effects of MIC-1 (1, 5, and 10 μM) and CEM (curcumin concentrations of 0.7, 3.5 or 7 μM) on LPS-stimulated NO production and viability of RAW 264.7 murine macrophages. A) NO production in MIC-1 and CEM-treated cells compared to Ctl without LPS and +LPS control. B) Viability of cells (MTT assay) treated with MIC-1 and CEM compared to no-treatment (NT) and Ctl cells. Data are the mean ± SD of 3 independent experiments for NO assay and 5 independent experiments for cell viability. Different letters indicate significant difference, one-way ANOVA followed by Tukey’s *post hoc* test.

A significant dose dependent reduction in NO production was observed in RAW 264.7 murine macrophages treated with MIC-1 and CEM without significantly affecting cellular viability (Fig 6). However, MIC-1 demonstrated a more dramatic and statistically significant effect than CEM at all concentrations. MIC-1 reduced LPS-induced NO production to basal levels by 72%, 91%, and 93% at concentrations of 1, 5, and 10 μM, respectively, corroborating results from a previous study [23] (Fig 6A). CEM reduced NO production by 5%, 13%, and 30% at the same concentrations (Fig 6A). In comparison, MIC-1 demonstrated a more dramatic and statistically significant effect than CEM at all concentrations. MIC-1 and CEM exerted no significant effect on cell viability with, determined by the MTT assay, compared to the control cells (Fig 6B).

#### Inflammatory gene expression

**Fig 7:**
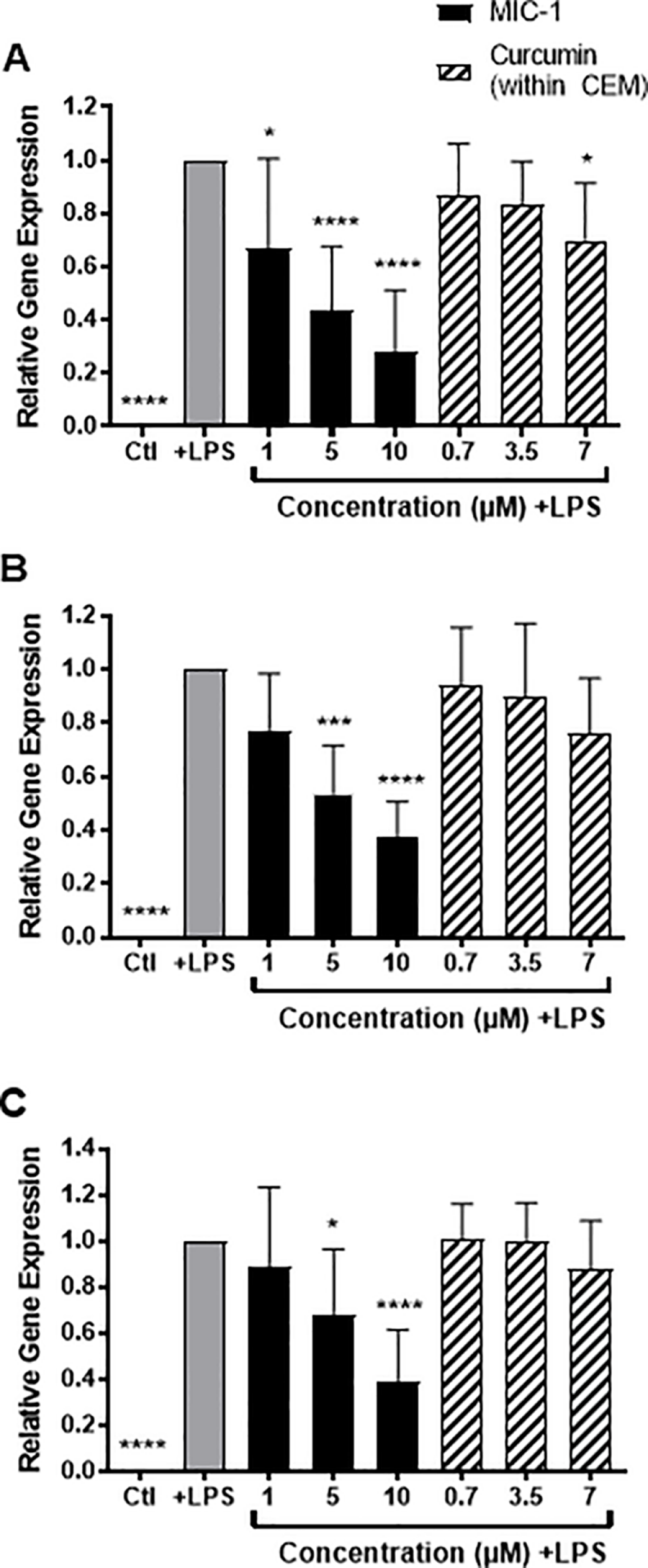
Effects of MIC-1 (1, 5, and 10 μM) and CEM (curcumin concentrations of 0.7, 3.5 or 7 μM) on LPS-stimulated pro-inflammatory gene expression in RAW 264.7 murine macrophages. Gene expression of A) iNOS, B) IL-1β, and C) IL-6 compared to Ctl without LPS and +LPS control. Data are the mean ± SD of 7 independent experiments. *P < 0.05 ***P < 0.001, ****P < 0.0001, one-way ANOVA followed by Dunnett’s *post hoc* test compared to +LPS control.

MIC-1 was significantly more effective at reducing iNOS expression than curcumin at all concentrations, with even 1 μM MIC-1 exhibiting higher bioactivity than 7 μM curcumin equivalent. MIC-1 treatment resulted in a significant, dose-dependent decrease in LPS-stimulated iNOS expression (Fig 7A). MIC-1, administered 2 h prior to LPS stimulation, reduced iNOS expression by 33%, 56%, and 72% at concentrations of 1, 5, and 10 μM, respectively, which is consistent with previously published data [23]. CEM also showed a dose-dependent reduction in iNOS expression by 13%, 16%, and 30% at curcumin concentrations of 0.7, 3.5 and 7 μM, respectively, however, the effect was only significant at 7 μM. Overall, MIC-1 at all concentrations significantly and more effectively decreased iNOS expression than CEM, with even the lowest concentration of MIC-1 exhibiting higher activity than the highest concentration of CEM.

MIC-1 was also significantly more effective at reducing the gene expression of pro-inflammatory cytokines IL-1β and IL-6 than curcumin at each concentration tested. MIC-1 induced a significant, dose-dependent reduction in IL-1β expression by 23%, 47%, and 62% at concentrations of 1, 5, and 10 μM, respectively compared with the untreated +LPS control (Fig 7B). CEM reduced IL-1β expression by 6%, 10%, and 24% at curcumin concentrations of 0.7, 3.5 and 7 μM but the result was not significant. MIC-1 significantly reduced IL-6 expression by 11%, 32%, and 61% at concentrations of 1, 5, and 10 μM, respectively (Fig 7C). CEM only showed non-significant reduction of IL-6 expression by 12% at curcumin concentration of 7 μM. Overall, MIC-1 was more potent than curcumin in reducing the gene expression of IL-1β and IL-6.

### *In vitro* antioxidant activity

**Fig 8:**
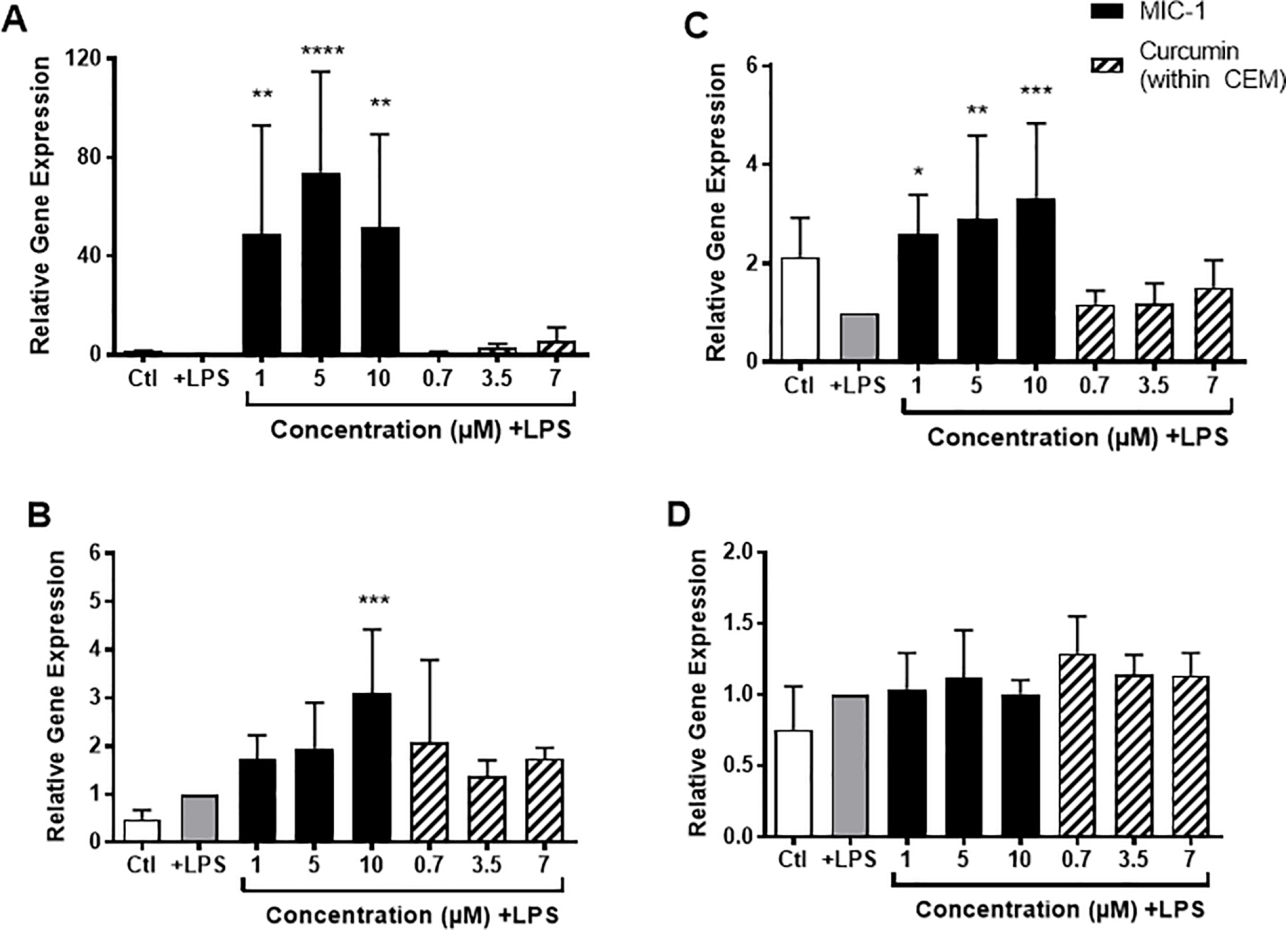
Effect of MIC-1 (1, 5, and 10 μM) and CEM (curcumin concentration of 0.7, 3.5 or 7 μM) on Nrf2-mediated detoxification gene expression in LPS-stimulated RAW 264.7 murine macrophages. Gene expression of A) NQO1, B) HO1, C) GSTP1, and D) NRF2 compared to Ctl without LPS and +LPS control. Data are the mean ± SD of 7 independent experiments. *P < 0.05, **P < 0.01, ***P < 0.001, ****P < 0.0001, one-way ANOVA followed by Dunnett’s *post hoc* test compared to +LPS control.

In murine macrophages, MIC-1 showed significant upregulation of all Nrf2 target genes (NQO1, HO1, GSTP1), while curcumin did not exhibit a significant effect on any gene target. MIC-1 significantly increased the expression of NQO1 by 49-fold, 74-fold, and 52-fold at concentrations of 1, 5 and 10 μM respectively. Meanwhile, CEM non-significantly increased NQO1 expression by 3-fold and 6-fold at curcumin concentrations of 3.5 and 7 μM, respectively (Fig 8A). MIC-1 increased HO1 expression by 2-fold and 3-fold at concentrations of 5 and 10 μM, respectively, with a statistically significant effect at the highest concentration (Fig 8B). CEM did not show a significant increase in HO1 expression compared to control. MIC-1 significantly increased GSTP1 expression about 3-fold at concentrations tested (1, 5 and 10 μM). However, CEM did not significantly affect GSTP1 expression (Fig 8C). The expression of the NRF2 gene was not significantly altered by MIC-1 or CEM treatments (Fig 8D).

## Discussion

We developed a simple, yet effective method for extracting moringa seeds to produce an extract that contains 38.9% of MIC-1. MIC-1 is a chemical and functional anolog of ITCs from cruciferous vegetables, but is more stable due to the attached sugar moiety. Most phytochemicals we identified in MSE have been previously reported from the moringa plant and some of their bioactivities reviewed [18,42–44]. Even though MIC-1 is, by far, the predominant phytochemical in MSE, we can not exclude the possibility that other phytochemical components may contribute to the bioactivity observed in the enriched extract. However, our *in vitro* results indicate that MIC-1 and MSE (containing equivalent MIC-1 concentrations) exhibited a comparable anti-inflammatory effect, strongly suggesting that MIC-1 is the likely predominant bioactive compound in MSE responsible for its anti-inflammatory properties, and its effect is not positively or negatively afffected by other MSE phytochemicals.

Many inflammatory processes, including acute inflammation in the carrageenan-induced rat paw edema model are thought to be biphasic, involving the sequential operation of multiple mediators. The initial phase (0–2 h) involves the release of bradykinin, histamine, and 5-hydroxy tryptamine (5-HT) while the second phase (2–6 h) involves an increased production of prostaglandins as well as the generation of NO via the iNOS pathway [45]. NO is also thought to play an important role in the mediation of acute and chronic inflammation. MIC-1 and other ITCs reduce NO production as well as iNOS gene expression [22,23,46]. The degree of reduction in the paw edema of animals treated with MSE was similar to that of the NSAID aspirin and was significantly more effective than CTE. One possible mechanism for the anti-inflammatory effect of MSE in the model could be the mediation of the NO pathway.

CTE was not effective in reducing the edema in the carrageenan-induced rat paw edema model. Decades of research have produced mixed results for the efficacy of turmeric/curcumin preparations and their anti-inflammatory properties. Reportedly, curcumin administered orally at 20–80 mg/kg showed inhibition of carrageenan-induced rat paw edema [47]. However, in another study using the same model, curcumin was reported to decrease the paw edema at low doses, while having a reversed effect at higher doses [48]. More recently, animals treated with turmeric extracts in this model showed significant decrease in paw edema in the first and third hours [49]. Low efficacy of either turmeric extracts or curcumin preparations observed in many *in vivo* studies, including our study, may be attributable to too high a dose [48], overall chemical instability of isolated compounds or poor systemic bioavailability [31]. MSE, on the other hand, was very effective at reducing inflammation when administered orally and its effect was comparable to that of the positive control, aspirin.

The inflammatory mediators (NO, iNOS, IL-1β and IL-6) are a part of the response observed in macrophages following LPS stimulation. MIC-1 treated cells showed significantly lower production of NO at nanomolar concentrations and reduced expression of iNOS, IL-1β and IL-6 at low micromolar concentrations (Figs 6 and 7), consistent with previous studies [23]. The levels of NO production observed were similar to those of the untreated control, and the extent of reduction was far greater than previously observed [22,23]. A prior study comparing MIC-1 to other cruciferous ITCs (sulforaphane and benzyl ITC) showed that MIC-1 was more effective at reducing NO production and iNOS expression at low micromolar concentrations [46]. Although MIC-1 showed inhibition of all the inflammatory markers tested, the greatest inhibition was observed in iNOS expression/NO production, which may be a primary target for MIC-1 anti-inflammatory effects. The expression of these inflammatory mediators are activated by NF-κB, however they can be differentially regulated by other transcription factors [50].

Nrf2 has been closely associated with the inflammatory response, either directly by preventing the expression of pro-inflammatory genes [51] or indirectly, by activating genes that suppress the accumulation of ROS and, in turn, increase inflammation [52]. ITCs induce expression of Nrf2 regulated genes, such as NQO1, HO1, and GSTP1, through binding to their antioxidant response element (ARE) promoter sequences [53]. Sulforaphane is considered one of the most potent inducers of Nrf2 and, subsequently, the phase II detoxification system [54,55]. It has been proposed that ITCs interact with Keap1, the protein that, under normal conditions, sequesters Nrf2 in the cytosol and targets it for degradation. ITCs therefore, are thought to release Nrf2 from Keap1, allowing it to translocate to the nucleus where it can induce the transcription of its target genes [56,57]. MIC-1 showed significant upregulation of all Nrf2 target genes tested in our study (NQO1, HO1, GSTP1), however no effect was observed on NRF2 gene expression (Fig 8). In corroboration with a previous study [58], these findings support the hypothesis that MIC-1 modulates Nrf2 activity at the proteomic level, likely through post-translational modification. Overall, the major moringa-derived constituent, MIC-1, is a powerful inhibitor of the pro-inflammatory mediators and inducer of Nrf2 regulated genes.

In contrast to MIC-1, CEM showed no significant effect on the anti-inflammatory or antioxidant mediators evaluated in this study except for a reduction in NO production and iNOS expression at a curcumin concentration of 7 μM. Our results do not support earlier studies that reported a comparatively more dramatic reduction in NO activity and iNOS expression [59,60] or the activation of the Nrf2 pathway by “curcumin” *in vitro* [8] and *in vivo* [29]. The limited *in vitro* anti-inflammatory and antioxidant activity of CEM observed could be due to the timing of the treatment or the low cell permeability of curcumin and curcuminoids as reported in the CaCo-2 cells drug permeability assay [61].

## Conclusion

MICs are naturally occurring anti-inflammatory compounds that are more stable than ITCs from cruciferous vegetables and are at least as efficacious. We developed an effective method that utilizes the *in situ* enzymatic conversion of GLSs to prepare a MIC-1-enriched (38.9% w/w) extract from moringa seeds. In addition to a very high MIC-1 content, MSE contains flavonoids, fatty acids, as well as fats, proteins, and carbohydrates. MSE was significantly more effective at reducing inflammation in a carrageenan-induced rat paw edema model than a commercially available CTE. The anti-inflammatory effect of MSE was comparable to that of the NSAID aspirin. MIC-1, the putative major anti-inflammatory component of MSE, significantly decreased inflammatory signaling (NO production, gene expression of iNOS, IL-1β, IL-6) and promoted detoxification (gene expression of NQO1, HO1, GSTP1) in LPS-stimulated murine macrophages. In comparison, CTEs, one of the most commonly used anti-inflammatory dietary supplement, showed very weak anti-inflammatory activities. These data validate the traditional uses of moringa as an anti-inflammatory botanical and highlight the potential of MIC-1-enriched moringa seed preparations to play a role in the prevention and treatment of chronic inflammatory conditions.

## Supporting information

S1 Fig Annotated ^1^H NMR spectrum of MIC-1. (DOCX)

S2 Fig Structure of glucosinolates and the related metabolites. (DOCX)

S3 Fig ^1^H NMR spectrum and quantitative analysis of CTE and CEM. (DOCX)

S1 Table Nutritional analysis of MSE. (DOCX)

S1 File Isothiocyanate-enriched moringa seed extract alleviates ulcerative colitis symptoms in mice. (PDF)
