# Supplementary material for: Biochemical characterization and anti-inflammatory properties of an isothiocyanate-enriched moringa (Moringa oleifera) seed extract
Source: PLoS One. 2017 Aug 8;12(8):e0182658. doi: 10.1371/journal.pone.0182658 (PMC5549737; doi:10.1371/journal.pone.0182658)
Supplement: S1 Fig — (DOCX) [file pone.0182658.s001.docx]

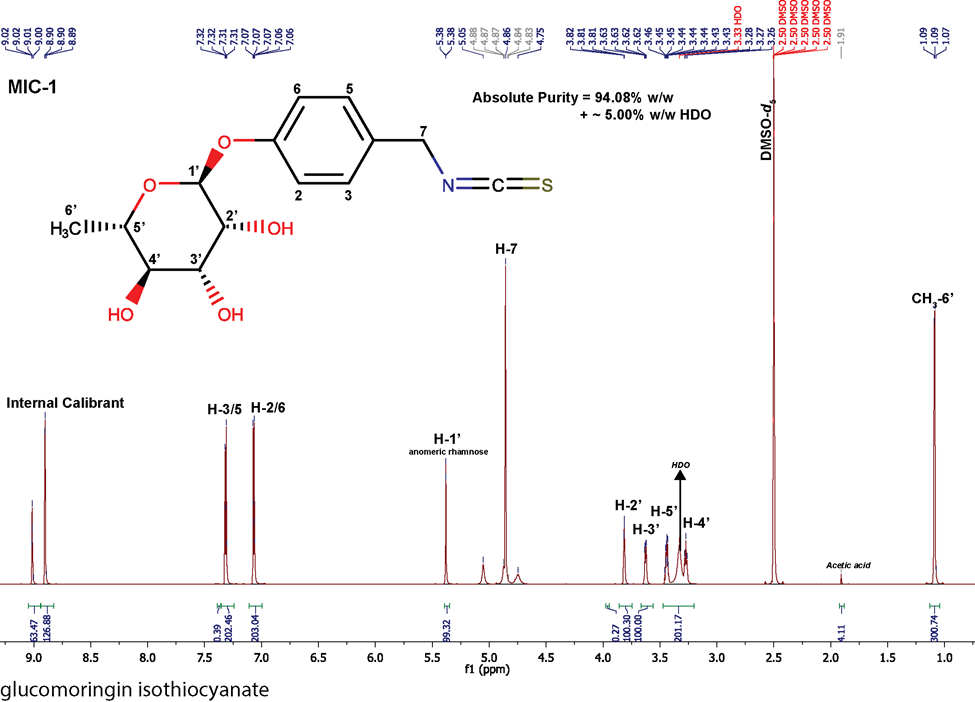


**S1 Fig. Annotated ^1^H NMR spectrum of MIC-1 together with the calculation of MIC-1 purity by the 100% method and the AIC method, and the HiFSA profile as PERCH.pms.**

The internal calibrant is: 3,5-dinitrobenzoic acid, DNBA (Fluka, TraceCERT, purity P = 99.54% w/w lot # BCBH8381V) at 11.6 mM in the sample. The analyzed 3 mm NMR tube (200 µL of DMSO-*d*_6_) contained 1.21 mg of MIC-1 and 0.49 mg of internal calibrant. The reported NMR data were in agreement with previously reported structural information (Muller C. et al. Phytochemistry 2015, 139-148, DOI:10.1016/j.phytochem.2015.08.007.
