# Supplementary material for: Biochemical characterization and anti-inflammatory properties of an isothiocyanate-enriched moringa (Moringa oleifera) seed extract
Source: PLoS One. 2017 Aug 8;12(8):e0182658. doi: 10.1371/journal.pone.0182658 (PMC5549737; doi:10.1371/journal.pone.0182658)
Supplement: S2 Fig — (DOCX) [file pone.0182658.s002.docx]

Glucosinolates and related metabolites

**S2 Fig. Structure of glucosinolates and the related metabolites; niazirin and niazimicin**
