# Supplementary material for: Biochemical characterization and anti-inflammatory properties of an isothiocyanate-enriched moringa (Moringa oleifera) seed extract
Source: PLoS One. 2017 Aug 8;12(8):e0182658. doi: 10.1371/journal.pone.0182658 (PMC5549737; doi:10.1371/journal.pone.0182658)
Supplement: S3 Fig — (DOCX) [file pone.0182658.s003.docx]

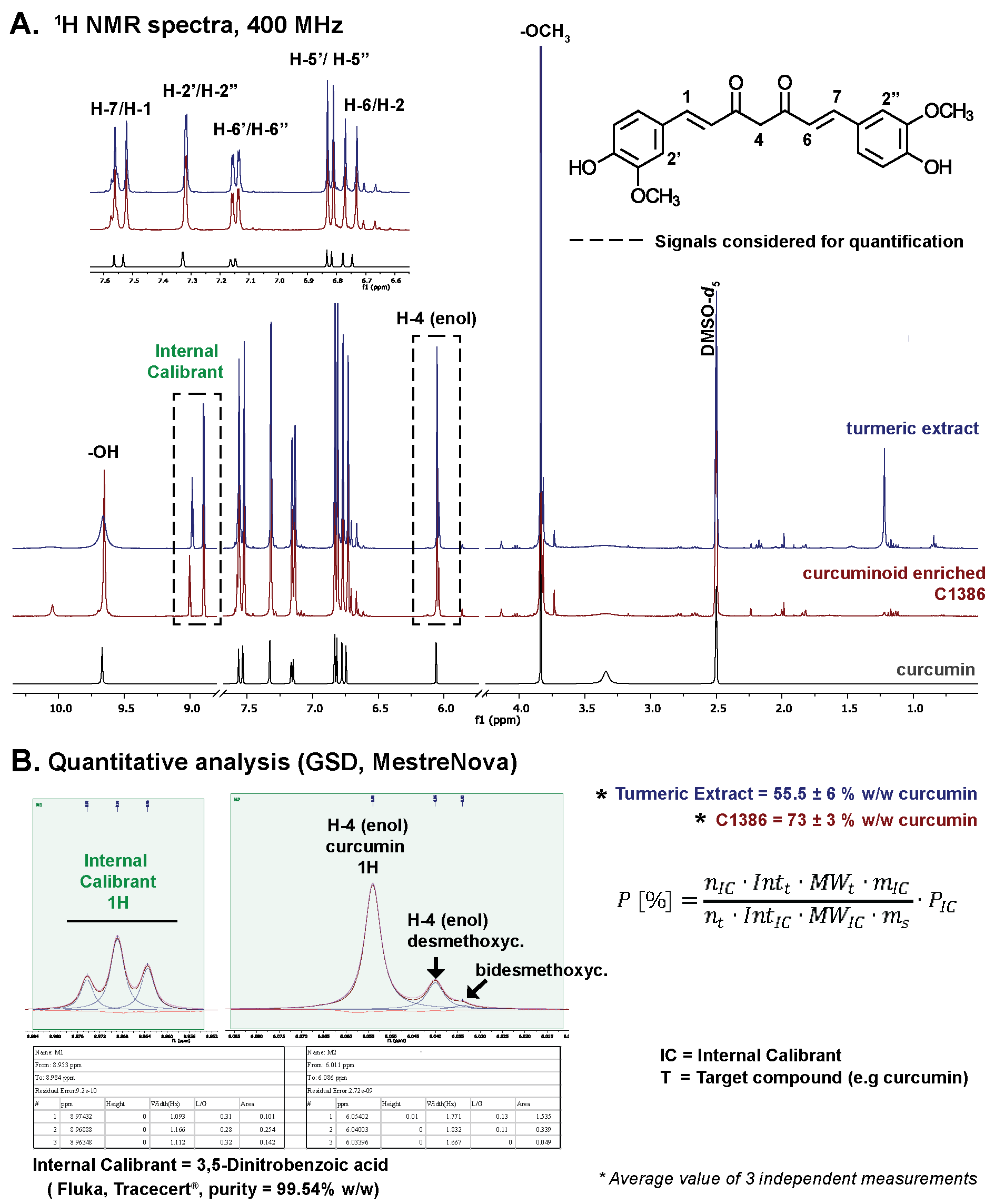


**S3 Fig. ^1^H NMR spectrum and quantitative analysis of CTE and CEM.**

### qHNMR of commercial curcumin preparations (CTE and CEM)

As described above, quantitative ^1^H NMR (qHNMR) analyses were performed using 3,5-dinitrobenzoic acid, DNBA (Fluka, TraceCERT, purity P = 99.54% w/w lot # BCBH8381V) as IC (11.6 mM in DMSO-*d*_6_). Three replicates of turmeric extract, CTE (Nature’s Bounty, Inc., Bohemia, NY) were prepared by weighing precisely ~ 8 mg of encapsulated powder which were diluted in exactly 300 µL of IC stock solution (final concentration 26.7 mg/mL). Three replicates of CEM (#C1386; Sigma-Aldrich, St. Louis, MO,) were precisely weighted (~3 mg) and diluted in exactly 200 µL of IC stock solution (final concentration 15 mg/mL). From these preparations, 200 μL aliquots were transferred with calibrated glass pipets into 3 mm standard NMR tubes. The 1D ^1^H NMR spectra were acquired at 298 K under quantitative conditions (qHNMR) using a 90° excitation pulse experiment on a Jeol ECZ 400 MHz equipped with a 5 mm probe. The probe was frequency tuned and impedance matched before each acquisition. For each sample, 96 scans and 4 dummy scans were recorded with the following parameters: pulse width of 6.87 μs (90°), spectral width of 25 ppm, relaxation delay of 30 s, receiver gain set to 52. The total duration of each ^1^H NMR acquisition was 55 min.

Off-line data processing was performed using the Mnova NMR software package as described above for MSE and MIC-1. Quantitative determination of curcumin, demethoxycurcumin, and bidesmethoxycurcumin (altogether constituting the curcuminoids) in each sample was determined through a non-quantum mechanical qHNMR method (See reference [33] in main article) utilizing the GSD module of the MNova software (see S3 Fig, and available data at [doi:10.7910/DVN/PRHUWB](http://dx.doi.org/10.7910/DVN/PRHUWB)) Accordingly, the amount of curcuminoids in CTE was calculated to be 68% w/w, and the curcumin level was found to be 55.5 ± 6% w/w. CEM contained 73 ± 3% of curcumin.
