# Supplementary material for: Biochemical characterization and anti-inflammatory properties of an isothiocyanate-enriched moringa (Moringa oleifera) seed extract
Source: PLoS One. 2017 Aug 8;12(8):e0182658. doi: 10.1371/journal.pone.0182658 (PMC5549737; doi:10.1371/journal.pone.0182658)
Supplement: S1 Table — (DOCX) [file pone.0182658.s004.docx]

# Nutritional Analysis of MSE

Table 1: Proximate analysis

| **Ingredient** | **%** |
| --- | --- |
| Moisture | 13.92 |
| Protein (crude) | 18.27 |
| Fat (crude) | 1.76 |
| Fiber (crude) | 0.60 |
| Ash | 4.01 |
| Carbohydrates | 22.54 |
| - Total Sugar | 45.4 |
| MIC-1 | 38.9 |

Table 2: Fatty acid profile

| **Fatty Acid Profile** | **C# : Dbl. Bonds** | **Relative Basis %** | **Sample Basis %** |
| --- | --- | --- | --- |
| Myristic | 14:0 | 0.20 | 0.01 |
| Pentadecanoic | 15:0 | 0.18 | 0.01 |
| Palmitic | 16:0 | 8.90 | 0.25 |
| Palmitoleic | 16:1 | 1.89 | 0.05 |
| Heptadecanoic | 17:0 | 0.16 | 0.00 |
| Stearic | 18:0 | 3.79 | 0.11 |
| Oleic | 18:1ω9 | 65.70 | 1.87 |
| Oleic | 18:1ω7 | 6.90 | 0.20 |
| Linoleic | 18:2ω6 | 1.53 | 0.04 |
| Linolenic | 18:3ω3 | 0.36 | 0.01 |
| Arachidic | 20:0 | 1.69 | 0.05 |
| Eicosanoic | 20:1ω9 | 1.58 | 0.04 |
| Behenic | 22:0 | 4.06 | 0.12 |
| Lignoceric | 24:0 | 0.64 | 0.02 |
| Other | n/a | 2.44 | 0.07 |
|  |  | 100.00 | 2.85 |
|  | Total % ω3 | 0.36 | 0.01 |
|  | Total % ω6 | 1.53 | 0.04 |
